# Membralin Selects Foreign Glycoproteins from the Endoplasmic Reticulum to Lysosomes for Degradation

**DOI:** 10.21203/rs.3.rs-6498082/v1

**Published:** 2025-06-23

**Authors:** Yong-Hui Zheng, Jing Zhang, Xiaoran Lu, Sunan Li, Tao Wang, Iqbal Ahmad

**Affiliations:** University of Illinois Chicago; Harbin Veterinary Research Institute, Chinese Academy of Agricultural Sciences; Harbin Veterinary Research Institute, Chinese Academy of Agricultural Sciences; Harbin Veterinary Research Institute; Harbin Veterinary Research Institute, Chinese Academy of Agricultural Sciences; Harbin Veterinary Research Institute

**Keywords:** Membralin, ERLAD, reticulophagy, class I fusion proteins, MAN1B1, TMEM259

## Abstract

The endoplasmic reticulum (ER) plays a central role in protein synthesis and folding. Membralin is a multi-pass membrane protein involved in ER-associated degradation (ERAD). Here, we demonstrate that Membralin assembles a protein degradation machinery across the ER membrane, specifically targeting class I fusion proteins expressed by major human viruses. Membralin interacts with MAN1B1 and p97/VCP through its luminal and cytoplasmic loops, respectively. Importantly, Membralin also contains an LC3-interacting region (LIR) in its cytoplasmic tail. The expression of these viral glycoproteins induces ER stress, prompting MAN1B1 to trim mannose residues extensively. Subsequently, Membralin recruits p97/VCP and initiate ER-phagy via its LIR, leading to degradation. This pathway specifically recognizes dense *N*-glycans and is selective, as it does not degrade misfolded domestic proteins. Collectively, our study reveals a cell-autonomous immunity inside the ER orchestrated by Membralin, underscoring its important role in the clearance of foreign glycoproteins to maintain cellular homeostasis.

## Introduction

The endoplasmic reticulum (ER) is essential for protein synthesis, producing both secreted and integral membrane proteins. It plays a critical role in ensuring that newly synthesized proteins are properly folded into their native conformations. When terminally misfolded proteins accumulate, the ER experiences stress, which can be mitigated by the ER quality control systems, including ER-associated degradation (ERAD) and ER-to-lysosome-associated degradation (ERLAD) ^1^.

Membralin/TMEM259, encoded by *TMEM259*, is crucial for motor neuron survival as it maintains optimal expression levels of nicastrin, a core component of the γ-secretase complex ^2^. It is a multi-pass membrane protein in the ER and implicated in alleviating ER stress by interacting with proteins in the ERAD network ^3^.

Mannosyl-oligosaccharide 1,2-α-mannosidase (MAN1B1) is a key enzyme in the ERAD process that specifically targets glycoproteins by co-opting with three ER degradation-enhancing α -mannosidase-like (EDEM) proteins. During glycoprotein folding in the ER, MAN1B1 primarily removes a single 1,2-α-linked mannose residue from Man9GlcNAc2 on *N*-glycan precursors, yielding Man8GlcNAc2. For terminally misfolded glycoproteins, MAN1B1 and EDEM proteins further trim two or three additional 1,2-α-linked mannose residues, producing Man(5–6)GlcNAc2. Glycoproteins with these low-mannose *N*-glycans are retrotranslocated and degraded via the ERAD pathway ^4^.

ERAD facilitates the retrotranslocation of misfolded proteins into the cytoplasm through the SEC61 translocon, an energy-dependent process driven by the AAA + ATPase p97/valosin-containing protein (VCP), ultimately resulting in degradation by cytosolic proteasomes. ERLAD directs misfolded proteins into specialized ER subdomains that detach from the ER and fuse with lysosomes for degradation. This process is mediated by 9 ER-phagy receptors, including FAM134B/RETREG1, FAM134B-2/RETREG1–2, FAM134A/RETREG2, FAM134C/RETREG3, TEX264, RTN3L, ATL3, CCPG1, and SEC62. These receptors are embedded in the ER membrane and are characterized by at least one cytosolic LC3-interacting region (LIR). FAM134B and RTN3L also contain a reticulon-homology domain (RHD) within the ER membrane.

Class I fusion proteins, such as human immunodeficiency virus type 1 (HIV-1) envelope glycoprotein (Env), influenza A virus hemagglutinin (HA), Ebola virus glycoprotein (GP), and severe acute respiratory syndrome coronavirus 2 (SARS-CoV-2) spike (S) protein, play crucial roles in the entry of these viruses ^5^. Although these proteins do not share sequence homology, they exhibit a common trimeric-hairpin structure with a central coiled coil covered by a dense glycan shield. Class I fusion proteins are expressed as type I transmembrane polypeptide precursors, folded in the ER, and cleaved by furin in the Golgi. We have previously shown that the expression of HIV-1-Env, EBOV-GP, and IFA-HA induces ER stress and undergoes MAN1B1-mediated degradation ^6–11^.

Here, we identify a protein degradation machinery across the ER membrane that specifically targets class I fusion proteins. This machinery is orchestrated by Membralin and includes MAN1B1 and p97/VCP, facilitating the degradation of these viral glycoproteins via ER-phagy. This process provides a crucial mechanism for cell-autonomous immunity within the ER.

## Results

### Human coronavirus spike proteins are MAN1B1 substrates.

SARS-CoV-2 (SARS2) infection induces ER stress, significantly contributing to COVID-19 cytopathic and inflammatory pathogenesis ^12–14^. During SARS-CoV (SARS1) infection, its spike protein plays a significant role in the induction of ER stress compared to other viral proteins ^15^. Thus, we investigated how SARS2-S activates UPR and triggers its relevant signaling events, including SARS1 and Middle East respiratory syndrome coronavirus (MERS) spike proteins as controls.

**Figure 1:**
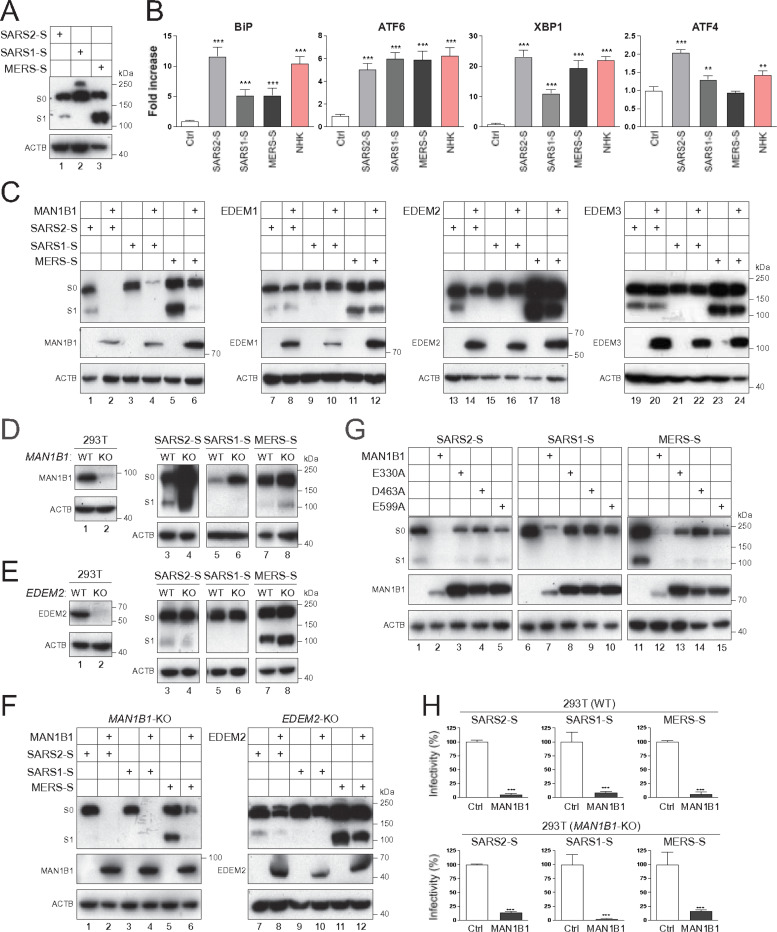
Error bars in (**B**) and (**H**) represent the standard error of measurements (SEMs) calculated from three experiments (n=3). *p<0.05, **p<0.01, ***p<0.001, ****p<0.0001, ns (not significant, p>0.05). Human coronavirus spike proteins are MAN1B1 substrates. (**A**) SARS1-S, SARS2-S, and MERS-S proteins with a FLAG-tag were expressed in HEK293T cells and detected by WB. The unprocessed S_0_ precursor and processed S_1_ subunit are indicated. b-actin (ACTB) was used as a loading control. (**B**) Coronavirus spike and NHK proteins were expressed with ER-stress luciferase-reporter vector pLightSwitch-BiP, p5ÁTF6-GL3, pXBP1u-FLuc, or pATF4-UTR-Fluc in HEK293T cells. Luciferase activity was measured and are presented as a relative value, with the activity in the presence of a control vector (Ctrl) set as 1. (**C**) Coronavirus spike proteins were expressed with MAN1B1, EDEM1, EDEM2, or EDEM3 with a HA-tag in HEK293T cells, and their expression was detected by WB. (**D**) Coronavirus spike proteins were expressed in HEK293T wild-type (WT) and *MAN1B1*-knockout (KO) cells, and their expression was compared by WB. (**E**) Coronavirus spike proteins were expressed in HEK293T WT and *EDEM2*-KO cells, and their expression was compared by WB. (**F**) Coronavirus spike proteins were expressed with MAN1B1 and EDEM2 in HEK293T *MAN1B1*-KO or *EDEM2*-KO cells, and their expression was compared by WB. (**G**) S proteins were expressed with MAN1B1 and its catalytic site-de cient mutants (E330A, D463A, E599A) in *MAN1B1*-KO cells, and their expression was analyzed by WB. (**H**) HIV-1 firefly luciferase reporter pseudovirions (PVs) expressing coronavirus spike proteins were produced in the presence of ectopic MAN1B1 from HEK293T WT or *MAN1B1*-KO cells. After infecting Huh7 cells with an equal number of PVs, viral infection was determined by measuring intracellular luciferase activities. Results are presented as relative values, with the activities in the presence of a control vector set as 100.

During ER stress, binding-immunoglobulin protein (BiP) is upregulated to serve as a master ER stress regulator, resulting in the activation of three ER transmembrane receptors: the protein kinase R (PKR)-like endoplasmic reticulum kinase (PERK), inositol-requiring enzyme type 1 (IRE1), and activating transcription factor 6 (ATF6). PERK activates a transcription factor ATF4, and IRE1 activates another transcription factor, X-box binding protein 1 (XBP1). As done previously, we used luciferase-based reporter assays to detect the upregulation and activation of BiP, XBP1, ATF4, and ATF6 ^6, 8^. As a positive control for ER stress, the terminally misfolded human Serpin A1 (SERPINA1)/alpha1-antitrypsin (AAT) variant, null (Hong Kong) (NHK), was used ^16^. SARS1-S, SARS2-S, MERS-S, and NHK were expressed with these different reporters, and levels of ER stress were determined. SARS1-S, SARS2-S, and MERS-S were expressed at similar levels, as detected by western blotting (WB) (Fig. 1A). As expected, NHK upregulated BiP and activated ATF6, XBP1, and ATF4 (Fig. 1B). Like NHK, SARS2-S also did so at comparable levels. SARS1-S and MERS-S strongly activated ATF6, but their upregulation and activation of BiP, XBP1, and ATF4 were significantly lower than SARS2-S and NHK. Thus, these coronaviruses activate ER stress, but the SARS2 activity is much broader and more potent.

Previously, we identified HIV-1-Env, IFA-HA, and EBOV-GP as MAN1B1 substrates for degradation ^6, 8, 10^. When SARS1-S, SARS2-S, or MERS-S were expressed with MAN1B1, EDEM1, EDEM2, and EDEM3, MAN1B1 reduced all these viral protein expressions, whereas EDEM1, EDEM2, and EDEM3 had little effect (Fig. 1C). We then knocked out *MAN1B1* and *EDEM2* (Fig. 1D, Fig. 1E). When SARS1-S, SARS2-S, and MERS-S were expressed in wild-type (WT) and these knockout (KO) cells, their expression was increased in *MAN1B1*-KO, but not *EDEM2*-KO cells. Next, ectopic MAN1B1 and EDEM2 were expressed with the individual spike proteins in KO cells. The MAN1B1 activity was restored, but EDEM2 had little activity (Fig. 1F).

MAN1B1 has three critical catalytic sites (E330, D463, and E599), which are conserved among class I α-mannosidases. When these residues were mutated to alanine, the MAN1B1 activity was disrupted, indicating that its enzymatic activity is required (Fig. 1G).

To demonstrate that this MAN1B1 activity is antiviral, HIV-1 luciferase-reporter pseudovirions (PVs) expressing SARS1-S, SARS2-S, and MERS-S were produced from HEK293T WT and *MAN1B1*-KO cells in the presence of ectopic MAN1B1, and their infectivity was determined by infection of Huh7 cells. MAN1B1 strongly inhibited the infectivity of all three PVs up to 40-fold (Fig. 1H).

### MAN1B1 targets coronavirus spike proteins to lysosomes.

We investigated how MAN1B1 affects the subcellular localization of coronavirus spike proteins by confocal microscopy.

**Figure 2:**
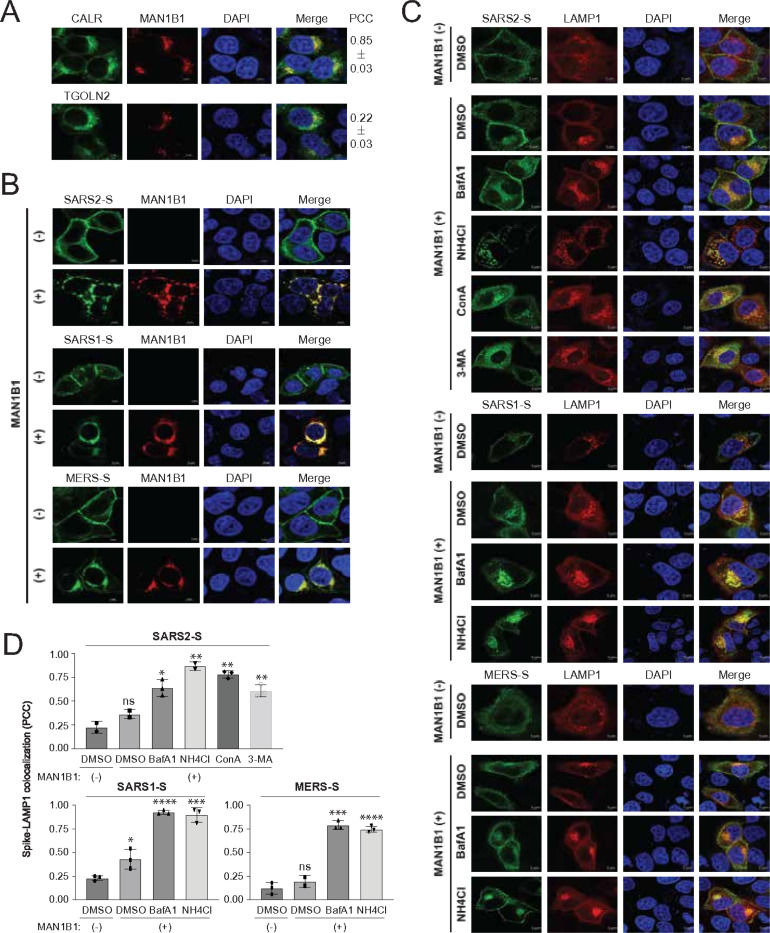
MAN1B1 targets coronavirus spike proteins to lysosomes. (**A**) MAN1B1 with a mCherry-tag and CALR or TGOLN2 with a GFP-tag were expressed in HeLa cells, and their colocalization was investigated by confocal microscopy (scale bar 5 μm). PCC, Pearson’s correlation coefficient. **(B)** Coronavirus spike proteins with a GFP-tag were expressed with MAN1B1-mCherry in HeLa cells and their subcellular localization was determined by confocal microscopy (scale bar 5 μm). **(C)** Coronavirus spike-GFP proteins were expressed with LAMP1-mCherry and MAN1B1 in HeLa cells. Cells were treated with 100 nM Bafilomycin A1 (BafA1), 20 mM NH_4_Cl, 20 nM concanamycin A (ConA), 10 mM 3-methyladenine (3-MA), or DMSO only. The co-localization of these spike proteins with LAMP1 was determined by confocal microscopy (scale bar 5 μm). **(D)** PCC values from (C) are calculated and shown. Error bars represent SEMs calculated from three experiments (n=3). *p<0.05, **p<0.01, ***p<0.001, ****p<0.0001, ns (not significant, p>0.05).

MAN1B1 with a mCherry-tag was expressed with the ER marker calreticulin (CALR), or the Golgi marker *trans*-Golgi network integral membrane protein 2 (TGOLN2) with a GFP-tag. MAN1B1 exhibited cytoplasmic distribution, colocalizing with both CALR and TGOLN2 (Fig. 2A). However, the Pearson’s correlation coefficient (PCC) of MAN1B1 with CALR was approximately four times higher than that with TGOLN2, confirming that MAN1B1 predominantly localizes to the ER.

When SARS1-S, SARS2-S, and MERS-S with a GFP-tag were expressed alone, they were predominantly found on the plasma membrane; however, when they were expressed with MAN1B1-mCherry, they were redistributed into cytoplasmic compartments and colocalized with MAN1B1 (Fig. 2B).

These spike proteins were then expressed with the lysosome marker, lysosomal-associated membrane protein 1 (LAMP1) with a mCherry-tag, and MAN1B1, followed by treatment with lysosomal/autophagy inhibitors NH_4_Cl, Bafilomycin A1 (BafA1), concanamycin A (ConA), and 3-methyladenine (3-MA). These spike proteins predominantly localized to the plasma membrane but were redistributed into the cytoplasm by the presence of MAN1B1, showing colocalization with LAMP1 (Fig. 2C). Notably, this colocalization was significantly promoted by treatments with these inhibitors (Fig. 2D), suggesting that spike proteins are degraded by autophagy/lysosomes.

### Class I fusion proteins are degraded in lysosomes independently of polyubiquitination.

**Figure 3:**
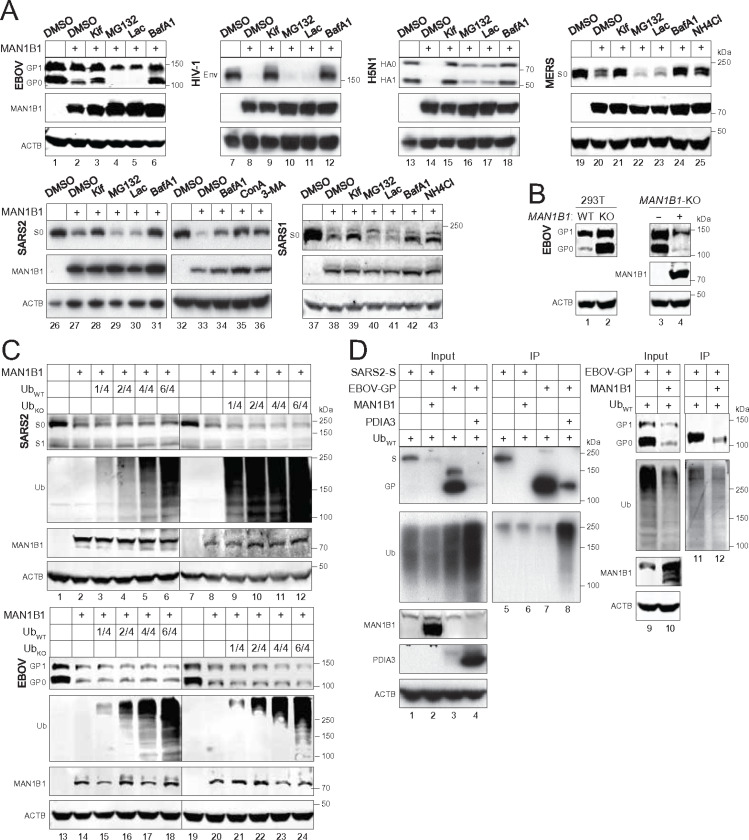
MAN1B1 targets class I fusion proteins for lysosomal degradation independently of polyubiquitination. **(A)** EBOV-GP, HIV-1-Env, H5N1-HA, SARS2-S, SARS1-S, and MERS-S were expressed with MAN1B1 in HEK293T cells and treated with 50 mM kifunensine (Kif), 20 mM MG132, 20 mM lactacystin (Lac), 100 nM BafA1, 20 mM NH_4_Cl, 20 nM ConA, or 10 mM 3-MA. Protein expression was determined by WB. **(B)** EBOV-GP was expressed in HEK293T WT and *MAN1B1*-KO cells, or, EBOV-GP was expressed in HEK293T MAN1B1-KO cells in the presence or absence of ectopic MAN1B1 expression. EBOV-GP expression was compared by WB. **(C)** SARS2-S and EBOV-GP were expressed with MAN1B1 in HEK293T cells in the presence of increasing amounts of WT ubiquitin (Ub_WT_) or its mutant that does not express any lysine residues (Ub_KO_). Protein expression was determined by WB. **(D)** SARS2-S and EBOV-GP with a FLAG-tag were expressed with Ub_WT_ and MAN1B1 or PDIA3 in HEK293T cells. Proteins were immunoprecipitated (IP) with anti-FLAG and analyzed by WB.

To detect protein degradation, class I fusion proteins, including EBOV-GP, HIV-1-Env, IFA (H5N1)-HA, SARS2-S, SARS1-S, and MERS-S were expressed with MAN1B1 in HEK293T cells and treated with different types of inhibitors, including class I α-mannosidase inhibitor kifunensine (Kif), proteasomal inhibitors MG132 and lactacystin (Lac), lysosomal inhibitors NH_4_Cl and BafA1, and autophagy inhibitors ConA and 3-MA. As expected, Kif blocked the MAN1B1 activity (Fig. 3A, lanes 3, 9, 15, 21, 28, 39), and neither MG132 nor Lac did so, indicating that they are not degraded in proteasomes. However, NH_4_Cl, BafA1, ConA, and 3-MA all blocked the MAN1B1 activity, indicating that they are degraded by autophagy/lysosomes. To further confirm the endogenous MAN1B1 activity, EBOV-GP was expressed again in WT and *MAN1B1*-KO cells. EBOV-GP expression was greatly increased in KO cells, which was countered by ectopic MAN1B1 (Fig. 3B).

Next, we determined whether polyubiquitination gets involved in this degradation process. We reported that disulfide-isomerase A3 (PDIA3) targets EBOV-GP for ER-phagy in a polyubiquitination-dependent manner ^8, 17^. When SARS2-S and EBOV-GP were expressed with MAN1B1 and increasing amounts of WT ubiquitin (Ub_WT_) or its lysine-free mutant (Ub_KO_), neither influenced their downregulation (Fig. 3C). SARS2-S and EBOV-GP were then expressed with MAN1B1 in the presence of Ub_WT_, and SARS2-S and EBOV-GP polyubiquitination were detected by immunoprecipitated (IP), including PDIA3 as a control. MAN1B1 and PDIA3 both decreased EBOV-GP expression (Fig. 3D, lanes 3, 4, 9, 10), but only PDIA3 promoted EBOV-GP polyubiquitination, whereas MAN1B1 did not (Fig. 3D, lanes 7, 8, 11, 12). Additionally, MAN1B1 did not induce SARS2-S polyubiquitination either (Fig. 3D, lanes 5, 6). Thus, MAN1B1 does not trigger SARS2-S and EBOV-GP polyubiquitination.

### Membralin is required for class I fusion degradation.

To identify the ER-phagy receptor, we knocked out *FAM134B*, *RTN3L*, *ATL3*, *SEC62*, *CCPG1*, and *TEX264* in HEK293T cells (**Fig. S1A**). Since FAM134B-2 is an N-terminally truncated isoform of FAM134B, this short isoform was also knocked out in this *FAM134B*-KO cell line. However, we found that MAN1B1 still decreased class I fusion protein expression in these KO cells (**Fig. S1B**). We also knocked out *FAM134C*, and excluded its involvement, too (**Fig. S1C**). Thus, MAN1B1 does not seem to engage with these known ER-phagy receptors to initiate this destruction.

**Figure 4:**
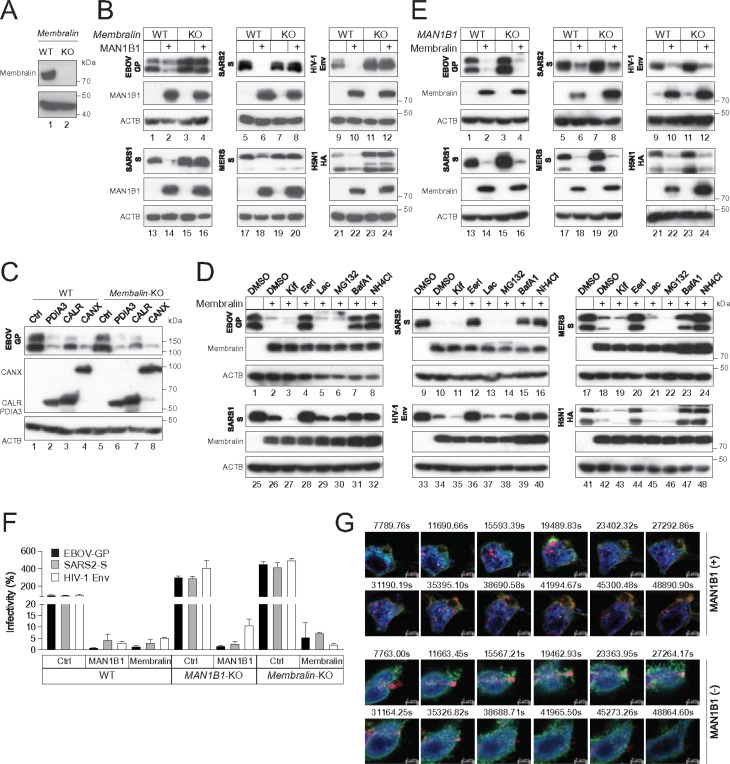
Membralin is required for class I fusion protein degradation. **(A)** Membralin was knocked out in HEK293T cells by CRISPR/Cas9, which is confirmed by WB. **(B)** Class I fusion proteins were expressed with MAN1B1 in HEK293T WT and *Membralin*-KO cells, and their expression was determined by WB. **(C)** EBOV-GP was expressed with indicated ER chaperones in HEK293T WT *Membralin*-KO cells, and their expression was determined by WB. **(D)** Class I fusion proteins were expressed with Membralin in HEK293T cells, and treated with 50 mM Kif, 10 mM Eerl, 20 mM Lac, 20 mM MG132, 100 nM BafA1, or 20 mM NH_4_Cl. Protein expression was determined by WB. **(E)** Class I fusion proteins were expressed with Membralin in HEK293T WT and *MAN1B1*-KO cells, and their expression was determined by WB. **(F)** PVs expressing HIV-1-Env, EBOV-GP, or SARS2-S were produced from HEK293T WT, *MAN1B1*-KO, and *Membralin*-KO cells in the presence of ectopic MAN1B1 or TMEM239 expression. Viral infectivity was determined in TZM-bI cells for PVs expressing HIV-1 Env, or in Huh7 cells for PVs expressing EBOV-GP or SARS2-S. Results are presented as relative values, with the activity in the presence of a control vector (Ctrl) set as 100. Error bars represent the SEMs calculated from three experiments. **(G)** SARS2-S-GFP, LAMP1-mCherry, and Membralin-BFP were expressed in HEK293T cells in the absence or presence of ectopic MAN1B1. Images were captured by Airyscan super-resolution microscopy every 5 min for 12.8 or 23.3 hours and are shown at indicated times. Scale bar, 5 mm.

Subsequently, we knocked out *Mebralin* in HEK293T cells (Fig. 4A). When class I fusion proteins were expressed alongside MAN1B1, the reduction of their expression was all blocked in this KO cell line (Fig. 4B, lanes 3, 4, 7, 8, 11, 12, 15, 16, 19, 20, 23, 24). However, PDIA3, CANX, and CALR continued to decrease EBOV-GP expression in these cells (Fig. 4C).

Next, we determined whether Membralin can target these viral proteins alone. Indeed, ectopic Membralin strongly decreased their expression (Fig. 4D, lanes 1, 2, 9, 10, 17, 18, 25, 26, 33, 34, 41, 42). This decrease was blocked by BafA1, NH_4_Cl, and the p97/VCP inhibitor Eeyarestatin I (EerI) but not by MG132, Lac, and Kif (Fig. 4D). To confirm the MAN1B1 independence, we expressed Membralin with class I fusion proteins in HEK293T WT and *MAN1B1*-KO cells. We found that Membralin still decreased the class I protein expression in this KO cell line (Fig. 4E).

Because Membralin was implicated in ERAD, we wondered whether the SEC61 complex, which serves as the translocon for retro-translocation during ERAD, is required for the Membralin activity. The SEC61 complex comprises abg subunits that form transmembrane channels where proteins are translocated across the ER membrane ^18^. The a subunit was silenced by siRNAs and the b subunit was knocked by CRISPR/Cas9 in HEK293T cells (**Fig. S2A, Fig. S2B**). When class I fusion proteins were expressed with MAN1B1 or Membralin, their expression was still inhibited in these silenced and KO cells (**Fig. S2A, Fig. S2C**). Thus, SEC61 translocon is not required for the MAN1B1 and Membralin activity.

To demonstrate that the MAN1B1-Membralin axis has antiviral activity, PVs expressing EBOV-GP, SARS2-S, and HIV-1-Env were produced from HEK293T WT, *MAN1B1*-KO, or *Membralin*-KO cells in the presence of ectopic MAN1B1 or Membralin expression. The infectivity of all these PVs was increased 3 to 4-fold when they were produced from *MAN1B1*-KO or *Membralin*-KO cells (Fig. 4F, compare Ctrl lanes in three cell lines). Conversely, their infectivity was decreased ~ 20-fold by ectopic MAN1B1 or Membralin (Fig. 4F). We previously reported the potent MAN1B1 antiviral activity to authentic IFA and HIV-1 ^6, 9, 10^. Collectively, these results demonstrate the broad antiviral activity of the MAN1B1-Membralin axis by targeting class I fusion proteins.

Furthermore, we used live-cell imaging to track the translocation of SARS2-S from the ER to lysosomes. SARS2-S-GFP, LAMP1-mCherry, and Membralin-BFP were expressed with MAN1B1 in HEK293T cells, and live cell images were captured by a super-resolution microscope every 5 min. Without MAN1B1, SARS2-S barely colocalized with Membralin and LAMP1 (Fig. 4G, **Fig. S3**), indicating successful egress of native SARS2-S from the ER for expression. In the presence of MAN1B1, SARS2-S was restricted into Membralin-positive domains that migrated into LAMP1-positive compartments time-dependent. These results confirm that MAN1B1 senses and recruits SARS2-S to the Membralin-positive ER-subdomain for degradation.

### The ER location of Membralin is determined by its 1st transmembrane domain.

**Figure 5:**
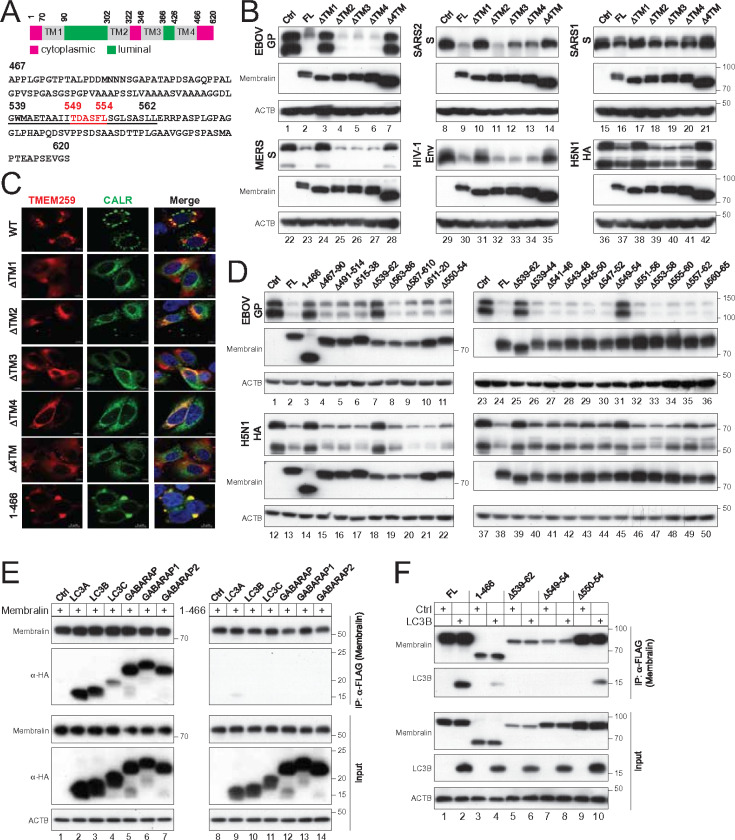
Membralin relies on different regions for ER-localization and functionality. **(A)** A schematic representation of Membralin is shown, featuring four transmembrane (TM) domains (grey), three cytoplasmic domains (pink), and two luminal domains (green), all indicated by their corresponding amino acid numbers. The sequence of the cytoplasmic tail (amino acids 467–620) is also displayed, with the region spanning amino acids 539–562 underlined and the ^549^TDASFL^554^ motif highlighted in red. **(B)** The indicated Membralin mutants were expressed with class I fusion proteins in HEK293T cells, and their expression was determined by WB. **(C)** CALR-GFP was expressed with indicated Membralin mutants with a FLAG-tag in HeLa cells. Cells were stained with anti-FLAG followed by Alexa Fluor 647-conjugated goat anti-mouse IgG, and their colocalization was determined by confocal microscopy (scale bar 5 μm). **(D)** The indicated Membralin mutants were expressed with EBOV-GP and H5N1-HA in HEK293T cells, and their expression was determined by WB. **(E)** Membralin and mutant 1–466 with a FLAG-tag were expressed with LC3/GABARAP family proteins with a HA-tag in HEK293T cells. Proteins were pulled down by anti-FLAG and analyzed by WB. **(F)** The indicated Membralin mutants with a FLAG-tag were expressed with LC3B with a HA-tag in HEK293T cells. Proteins were pulled down by anti-FLAG and analyzed by WB.

Membralin consists of 620 amino acids (aa) and features four transmembrane (TM) domains, three cytoplasmic domains, and two luminal domains (Fig. 5A). We initially created five Membralin deletion mutants by removing each TM domain individually (ΔTM1, ΔTM2, ΔTM3, ΔTM4) and an additional mutant that removed all four TM domains (Δ4TM). When expressed alongside class I fusion proteins, unlike the full-length (FL) Membralin, ΔTM2, ΔTM3, and ΔTM4, neither ΔTM1 nor Δ4TM reduced their expression (Fig. 5B).

Next, we compared the subcellular localization of these mutants using confocal microscopy, with CALR as the ER marker. We found that ΔTM2, ΔTM3, and ΔTM4 localized correctly within the ER, whereas ΔTM1 and Δ4TM did not (Fig. 5C). These results demonstrate that TM1 is essential for the ER localization, and importantly, this localization is critical for its activity.

### The cytoplasmic tail of Membralin interacts with LC3/GABARAP family proteins.

To screen for active motifs, we constructed additional Membralin deletion mutants. Mutant 1–466, which possesses the entire cytoplasmic tail deletion, localized to the ER (Fig. 5C), but failed to reduce the expression of EBOV-GP and H5N1-HA (Fig. 5D, lanes 3 and 15), indicating the functional importance of this cytoplasmic tail.

We created six 24-aa-deletion mutants (Δ467–490, Δ491–514, Δ515–538, Δ539–562, Δ563–586, and Δ587–610) and one 10-aa-deletion mutant (Δ611–620) to screen the 467–620 region. When these seven mutants were expressed alongside EBOV-GP and H5N1-HA, only mutant Δ539–562 lost the activity (Fig. 5D, lanes 7, 18). To further narrow down this 24-aa region, we constructed 11 overlapping 6-aa-deletion mutants, including Δ539–544, Δ541–546, Δ543–548, Δ545–550, Δ547–552, Δ549–554, Δ551–556, Δ553–558, Δ555–560, Δ557–562, and Δ560–565. When expressed with EBOV-GP and H5N1-HA, only mutant Δ549–554 lost the activity (Fig. 5D, lanes 31, 45).

The 549–554 region contains 6 residues TDASFL (Fig. 5A). To gain further insight, we created mutant Δ550–554 by deleting the last ve residues DASFL and found that it retained the activity (Fig. 5D, lanes 11, 22). Because mutants Δ545–550, Δ547–552, and Δ551–556 contain partial deletions of these six residues but still exhibit activity, these results demonstrate that the complete deletion of ^549^TDASFL^554^ must occur to inactivate Membralin’s activity.

Next, we tested whether this ^549^TDASFL^554^ domain functions as an LC3-interacting region (LIR), which is characteristic of ER-phagy receptors. The LIR is a short peptide sequence that serves as a binding site for LC3/GABARAP proteins, which are crucial for autophagy. We expressed Membralin and mutant 1–466 alongside LC3A, LC3B, LC3C, GABARAP, GABARAP1, and GABARAP2 to assess their interactions through immunoprecipitation. Membralin successfully pulled down all these autophagy proteins, whereas 1–466 did not (Fig. 5E). We then expressed Δ539–562, Δ549–554, and Δ550–554 with LC3B and repeated this experiment. Δ550–554 was expressed at a similar level to the FL protein but showed significantly weaker binding to LC3B (Fig. 5F, lanes 2, 10). On the other hand, 1–466, Δ539–562, and Δ549–554 were all expressed at similar lower levels, and none of them bound to LC3B (Fig. 5F, lanes 4, 6, 8). Collectively, these results identify ^549^TDASFL^554^ as a critical motif for LC3-binding.

### Membralin recruits VCP via its cytoplasmic loop.

**Figure 6:**
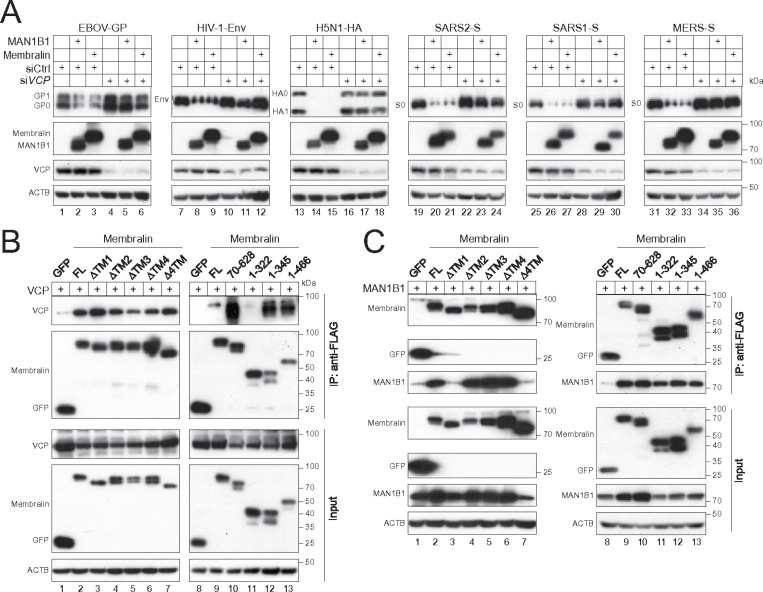
Membralin recruits VCP and MAN1B1 via different regions. **(A)** Class I fusion proteins were expressed alongside MAN1B1 or Membralin in HEK293T cells in the presence of siRNAs targeting *VCP* or a control (Ctrl). Protein expression levels were assessed by WB. **(B)** VCP-HA was co-expressed with the indicated Membralin deletion mutants or GFP as a control in HEK293T cells. Proteins were pulled down by anti-FLAG beads. Proteins in the pulldown samples (IP) and cell lysate (Input) were detected by WB. **(C)** MAN1B1-HA was expressed with the indicated Membralin mutants and GFP with a FLAG-tag in HEK293T cells. Proteins were pulled down by anti-FLAG beads. Proteins in the pulldown samples (IP) and cell lysate (Input) were detected by WB.

The Membralin activity is blocked by the VCP inhibitor EerI (Fig. 4D, lanes 4, 12, 20, 28, 36, 44), indicating an involvement of this AAA + ATPase. To further confirm this, we silenced *VCP* in HEK293T cells by siRNAs and tested the MAN1B1 and Membralin activity. The *VCP* siRNA disrupted the MAN1B1 and Membralin activity class I fusion proteins, but a control siRNA did not (Fig. 6A).

The four TM domains of Membralin are linked by one large and one small luminal loop (aa 91–301, 367–425, Fig. 5A, green) and a relatively shorter cytoplasmic loop (aa 323–345, Fig. 5A, middle pink). To understand how VCP interacts with Membralin, we constructed another three Membralin deletion mutants to express aa 70–628, 1–322, or 1–345. Together with those five TM domain deletion mutants and mutant 1–466, they were expressed with VCP in HEK293T cells, and their interactions were determined by immunoprecipitation. The FL Membralin and its five TM deletion mutants pulled down VCP, whereas GFP did not, indicating that the Membralin-VCP binding does not depend on TM domains (Fig. 6B, lanes 1 to 7). In addition, 70–628, 1–345, and 1–466 were also bound to VCP, whereas 1–322 did not (Fig. 6B, lanes 10–13), indicating that Membralin recruits VCP via its cytoplasmic loop to destroy class I fusion proteins.

### Membralin recruits MAN1B1 via its large luminal loop.

We used the same mutants to understand the Membralin-MAN1B1 interaction by immunoprecipitation. All these mutants successfully pulled down MAN1B1, except for ΔTM1 and Δ4TM (Fig. 6C, lanes 3, 7). Since these two mutants do not localize to the ER, these results confirm that the MAN1B1-Membralin interaction must occur within the ER. Additionally, 1–466 was able to pull down MAN1B1, indicating that this interaction does not require the cytoplasmic tail of Membralin. Furthermore, the continued interaction of 1–322 and ΔTM2 with MAN1B1 further establishes that MAN1B1 specifically binds to the large luminal loop of Membralin (aa 90–302).

### Membralin is not required for degradation of domestic misfolded proteins.

Mutations on human genes encoding AAT ^19^, collagen α−1(II) chain (COL2A1) ^20^, Niemann-Pick type C1 (NPC1) ^21^, and dysferlin (DYSF) ^22, 23^ can cause their misfolding in the ER, leading to degradation by ERQC and various genetic diseases.

MAN1B1 targets AAT variant NHK to ERAD ^16, 24^, but targets variant Z (ATZ) to ERLAD ^25^. NPC1 variant I1061T is targeted to ERAD ^26^, or ERLAD, via the ER-phagy receptor FAM134B ^27^. CANX also targets COL2A1 variant R989C/G1152D to ERLAD via FAM134B ^28^. The DYSF variant L1341P is targeted to either ERAD or ERLAD ^29^. Additionally, the autocrine motility factor receptor (AMFR) targets human CD3δ (CD3D) to ERAD ^30^, and nicastrin (NCSTN) in the γ-secretase complex is also targeted to ERAD ^2^. We collectively tested whether the MAN1B-Membralin axis could target these human proteins.

**Figure 7:**
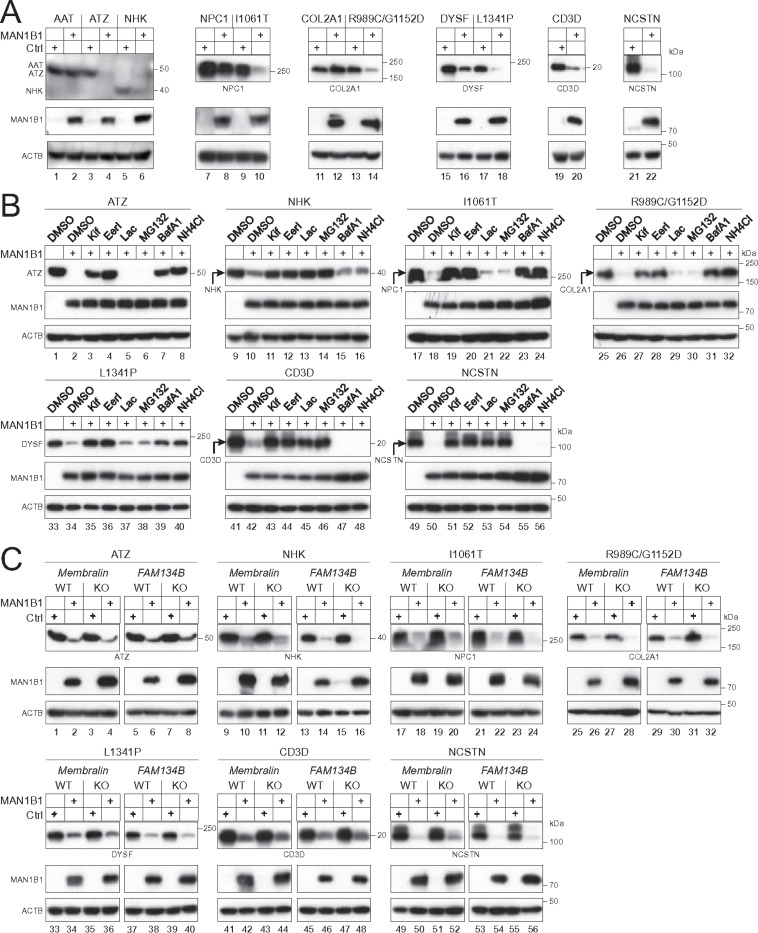
The Membralin-MAN1B1 axis does not target well-defined human misfolded or aggregated proteins. **(A)** AAT and its variants ATZ and NHK, NPC1 and its variant I1061T, collagen alpha-1(II) chain (CO2A1) and its variant R989C/G1152D, dysferin (DYSF) and its variant L1341P, CD3d (CD3D), and nicastrin (NCSTN) were expressed with MAN1B1 in HEK293T cells and their expression was determined by WB. **(B)** The indicated misfolded and aggregated proteins were expressed with MAN1B1 in HEK293T cells. Cells were treated with 50 mM Kif, 10 mM EerI, 20 mM Lac, 20 mM MG132, 100 nM BafA1, or 20 mM NH_4_Cl. Protein expression was determined by WB. **(C)** The indicated misfolded and aggregated proteins were expressed with MAN1B1 in HEK293T WT, *Membralin*-KO, and *FAM134B*-KO cells. Protein expression was determined by WB.

First, we expressed AAT and its variants ATZ and NHK, NPC1 and its variant I1061T, COL2A1 and its variant R989C/G1152D, DYSF and its variant L1341P, CD3δ, and NCSTN with MAN1B1 in HEK293T cells. MAN1B1 effectively decreased the ATZ, NHK, 1061T, R989C/G1152D, L1341P, CD3δ, and NCSTN expression (Fig. 7A, lanes 4, 6, 10, 14, 18, 20, 22), but much less effectively the WT AAT, NPC1, COL2A1, and DYSF expression (Fig. 7A, lanes 2, 8, 12, 16).

Second, we determined how these misfolded variants, CD3δ, and NCSTN, are degraded. All their decreases by MAN1B1 were blocked by Kif and EerI (Fig. 7B, lanes 3, 4, 11, 12, 19, 20, 27, 28, 35, 36, 43, 44, 51, 52). The decrease of NHK, CD3δ, and NCSTN was blocked by Lac and MG132, indicating that they are targeted to ERAD (Fig. 7B, lanes 13, 14, 45, 46, 53, 54). The decrease of ATZ, I1061T (NPC1), R989C/G1152D (COL2A1), and L1341P (DYSF) was blocked by BafA1 and NH_4_Cl, indicating that they are targeted to ERLAD (Fig. 7B, lane 7, 8, 23, 24, 31, 32, 39, 40).

Third, we determined whether Membralin is required. These proteins were expressed with MAN1B1 in HEK293T WT and *Membralin*-KO cells. Because FAM134B was required for R989C/G1152D (COL2A1) and I1061T (NPC1) degradation via ERLAD, the *FAM134B*-KO cell line was also included. Notably, none of their degradation was blocked in *Membralin*-KO and *FAM134B*-KO cells (Fig. 7C).

### Selection of ER-phagy receptors.

Surprisingly, FAM134B is not required for MAN1B1-mediated degradation of I1061T (NPC1) and R989C/G1152D (COL2A1) (Fig. 7C, lanes 23, 24, 31, 32). To understand how ER-phagy receptors are selected, ATZ, I1061T (NPC1), R989C/G1152D (COL2A1), and L1341P (DYSF) were expressed with MAN1B1 in HEK293T WT cells and six ER-phagy receptor KO cell lines. MAN1B1 still effectively decreased their expression in these KO cells, indicating that these ER-phagy receptors are not required for the MAN1B1 activity (**Fig. S4**).

**Figure 8:**
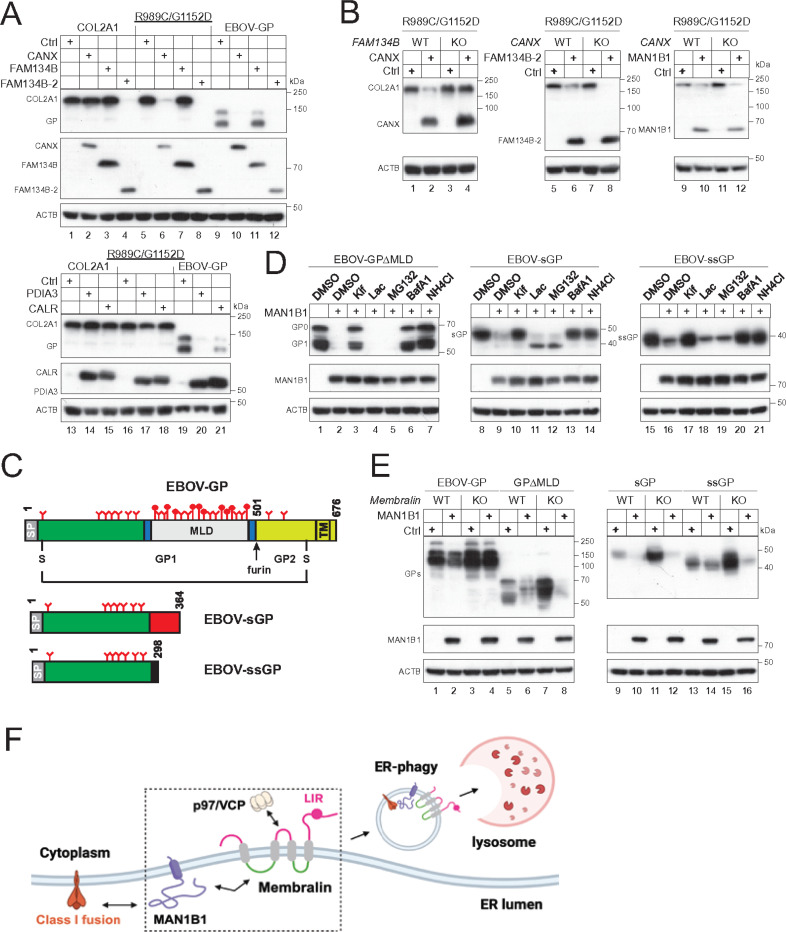
Multiple factors determine the specific degradation. **(A)** COL2A1, its variants R989C/G1152D, and EBOV glycoprotein (GP) were co-expressed with calnexin (CANX), protein disulfide isomerase A3 (PDIA3), calreticulin (CALR), FAM134B, and FAM134B-2 in HEK293T cells. Protein expression levels were assessed by WB. **(B)** The R989C/G1152D variant was expressed with CANX, FAM134B-2, or MAN1B1 in HEK293T wild-type (WT), *FAM134B*-KO, or *CANX*-KO cells. Protein expression was again evaluated by WB. **(C)** A schematic representation of EBOV glycoproteins is shown, including the structural glycoprotein (GP), secreted glycoprotein (sGP), and soluble glycoprotein (ssGP). The structural glycoprotein is processed into GP_1_ and _GP2_ by furin and linked by a disulfide bond. The letter “Y” indicates *N*-glycosylation sites, while indicates *O*-glycosylation sites. MLD refers to the mucin-like domain, and TM indicates the transmembrane domain. **(D)** The indicated EBOV glycoproteins were expressed alongside MAN1B1 in HEK293T cells and treated with the specified inhibitors. Protein expression levels were determined by WB. Indicated EBOV glycoproteins were expressed with MAN1B1 in HEK293T cells and treated with indicated inhibitors. Protein expression was determined by WB. **(E)** The indicated EBOV glycoproteins were expressed with MAN1B1 in HEK293T WT and *Membralin*-KO cells, and protein expression was assessed by WB. **(F)** A proposed model for the degradation of class I fusion proteins by Membralin. During productive viral infection, heavily glycosylated class I fusion proteins are recognized by MAN1B1, which catalyzes their extensive demannosylation in the ER. The Membralin-MAN1B1 complex then targets these low-mannose viral glycoproteins to an ER subdomain, from which they are detached via p97/VCP and engulfed in lysosomes via Membralin LIR for ER-phagy.

To further explore this mechanism, we investigated the degradation of COL2A1 and its variant R989C/G1152D in the context of CANX and FAM134B. CANX, CALR, and PDIA3 were also included because they degrade EBOV-GP via ERLAD. Consistently, CANX, CALR, and PDIA3 decreased EBOV-GP expression (Fig. 8A, lanes 10, 20, 21), as previously reported ^8, 17^, and CANX decreased R989C/G1152D but not COL2A1 expression (Fig. 8A, lanes 2, 6), as reported ^28^. FAM134B did not decrease any COL2A1, R989C/G1152D, and EBOV-GP expression (Fig. 8A, lanes 3, 7, 11), but FAM134B-2 decreased all these protein expression (Fig. 8A, lanes 4, 8, 12).

Additionally, CANX did not decrease R989C/G1152 expression in *FAM134B*-KO cells (Fig. 8B, lanes 3, 4), confirming that FAM134B is the ER-phagy receptor for this degradation ^28^. FAM134B-2 and MAN1B1 still decreased R989C/G1152D expression in *CANX*-KO cells, indicating that their activity is independent of CANX (Fig. 8B, lanes 5–12). These results demonstrate that the selection of ER-phagy receptor is determined by both effector (MAN1B1, CANX, FAM134B-2) and client proteins (host and viral proteins), highlighting the speci city of the Membralin-MAN1B1 axis in class I fusion protein targeting.

### The Membralin-MAN1B1 axis senses dense glycans.

In addition to the structural GP, EBOV expresses soluble GP (sGP) and small soluble GP (ssGP) via RNA editing ^8, 17^, all sharing the same N-terminal domain (Fig. 8C). EBOV-GP is heavily glycosylated, particularly in the mucin-like domain (MLD) ^31^. It has 17 *N*-glycosylation sites, 8 of which are in MLD. In addition, MLD contains over 80 *O*-glycosylation sites, resulting in higher GP_1_ molecular weight than the precursor GP_0_ (Fig. 3A, lanes 1–6; Fig. 3B, lanes 1–4). To understand how glycans contribute to GP degradation, we tested the degradation of GP deletion mutant GPΔMLD that does not express MLD, along with sGP and ssGP. MAN1B1 decreased their expression, which was blocked by Kif, BafA1, and NH_4_Cl, but not Lac and MG132 (Fig. 8D). Thus, MAN1B1 could still target these less-glycosylated GP to lysosomes for degradation. However, when EBOV-GP, GPΔMLD, sGP, and ssGP were expressed with MAN1B1 in *Membralin*-KO cells, only the decrease of FL EBOV-GP was blocked, whereas the others were not (Fig. 8E, lanes 3, 4). Thus, dense glycans are required for Membralin-MAN1B1 to target EBOV-GP for degradation.

## Discussion

Membralin has been implicated as a critical component of the ERAD pathway. It forms a complex with the E3 ubiquitin ligase RNF185 and the UBL domain-containing proteins TMUB1 and TMUB2 on the ER membrane. This complex works in conjunction with the cytosolic ubiquitin ligase UBE3C and p97/VCP to facilitate the degradation of membrane substrates ^32^. However, inconsistent findings have been reported regarding Membralin-mediated degradation of NCSTN, NHK, and CD3δ ^2, 32^. Our results also indicate that Membralin is not necessary for MAN1B1-mediated degradation of NHK, CD3δ, and NCSTN in proteasomes. Consequently, the role of Membralin in ERAD warrants further investigation.

We find that Membralin is essential not only for MAN1B1-initiated ERLAD but also for directly initiating the degradation process. While both MAN1B1 and PDIA3 can trigger the degradation of EBOV-GP via ERLAD, the precise mechanisms appear to differ. For instance, MAN1B1 does not induce polyubiquitination of its substrate, whereas PDIA3 does, consistent with our previous findings that PDIA3’s activity is dependent on RNF185 ^17^. Therefore, Membralin’s activity should not rely on RNF185. ERLAD clients are thought to be segregated into ER subdomains rather than being retrotranslocated across the ER membrane for engulfment ^33^. In line with this, we find that the SEC61 translocon is not required for the degradation of class I fusion proteins. However, Membralin recruits p97/VCP through its cytoplasmic loop, and p97/VCP is essential for its activity. While p97/VCP serves as a critical energy provider for ERAD, it also performs diverse cellular functions, including membrane remodeling ^34^, which we speculate facilitates the formation and detachment of ER subdomains. Like other known ER-phagy receptors, Membralin contains an LIR in its cytoplasmic tail, which should promote the degradation of class I fusion proteins through ER-phagy.

We find that Membralin recruits MAN1B1 via its luminal loop to specifically target class I fusion proteins rather than known misfolded proteins or aggregates. During productive infection, viruses exploit host cells to produce abnormally high levels of class I fusion proteins, overwhelming the ER’s folding machinery and inducing ER stress. Class I fusion proteins possess distinctive features that facilitate their recognition and clearance as foreign proteins. They are heavily glycosylated, primarily with *N*-glycans and fewer *O*-glycans, except for EBOV-GP, where *O*-glycans predominate. For instance, HIV-1 Env (gp120) undergoes extensive glycosylation, with approximately half of its molecular weight attributed to glycans ^35^. While this dense glycan shield obscures key epitopes and aids immune evasion, it may also act as a non-host marker for being detected by MAN1B1. Our findings consistently demonstrate that significantly reduced glycosylation levels prevent EBOV-GP from being targeted by the Membralin-MAN1B1 axis. However, less glycosylated EBOV-GP can still undergo degradation via ERLAD through an alternative, unidentified mechanism. These results underscore glycosylation levels as a critical determinant for targeting by this protein complex.

We confirm that, unlike NHK, the AAT polymeric variant ATZ is directed to ERLAD rather than ERAD, consistent with previous findings ^36^. It has been proposed that misfolded large polypeptide aggregates resist ERAD due to the challenges of translocating through the SEC61 translocon and entering the proteasomes. In alignment with this, we observe that misfolded variants of NPC1, COL2A1, and DYSF are targeted to ERLAD but not ERAD. Class I fusion proteins, with their large molecular weights (exceeding 70 kDa) and trimeric structures, likely follow the same pattern, as their size and aggregation render them suitable candidates for ERLAD rather than ERAD.

MAN1B1 is well-established in targeting the luminal substrate NHK to ERAD. Our findings further reveal that it also targets the membrane substrate CD3δ and another luminal substrate, NCSTN, through the same mechanism, demonstrating that MAN1B1 recognizes both ERAD luminal (ERAD-L) and ERAD membrane (ERAD-M) substrates. Moreover, MAN1B1 directs other misfolded human proteins, including AAT, NPC1, COL2A1, and DYSF variants (ATZ, I1061T, R989C/G1152D, and L1341P), to ERLAD. This degradation pathway operates independently of known ER-phagy receptors or Membralin, implying that MAN1B1 interacts with other unidentified ER-phagy receptor(s) for these host proteins. We also confirmed that CANX facilitates the degradation of the R989C/G1152D variant in a FAM134B-dependent manner ^28^. These findings suggest that the selection of an ER-phagy receptor is dictated by both the effector (MAN1B1, CANX) and the speci c client proteins.

In summary, we nd that Membralin orchestrates a protein degradation machinery across the ER membrane by recruiting MAN1B1 and p97/VCP, selectively targeting foreign glycoproteins via ER-phagy (Fig. 8F). This mechanism represents a new arm of cell-autonomous immunity, playing a significant role in defending eukaryotic cells against viral infections.

## Materials and Methods

### Antibodies and inhibitors.

Commercial reagents include: kifunensine, eeyarestatin I, MG132, Lactacystin (Lac), Concanamycin A (ConA), 3-Methyladenine (3-MA), and ammonium chloride (NH_4_Cl) (Sigma-Aldrich, K1140, E1286, C2211, L6785, C9705, M9281, A9434); Bafilomycin A1 (BafA1, Santa Cruz Biotechnology, sc-201550); mouse monoclonal anti-Myc (CST, 2276S); rabbit polyclonal anti-SQSTM1, mouse monoclonal anti-FLAG and anti-HA (Sigma-Aldrich, P0067, F3165, H3663); mouse monoclonal anti-ACTB/β-actin (CST, 3700S); mouse monoclonal anti-ERManI/MAN1B1 (Novus, NBP2–13167); rabbit polyclonal anti-FAM134B-2 and anti-Membralin (Gene Tex, GTX46621, GTX118618); rabbit polyclonal anti-ATL3, anti-FAM134B, anti-CCPG1, anti-TEX264, and anti-TOLLIP (Proteintech, 16921–1-AP, 21537-AP, 13861–1-AP, 25858–1-AP, 11315–1-AP); rabbit monoclonal anti-SEC62 and rabbit polyclonal anti-EDEM2 (Abcam, ab137022, ab181218); rabbit polyclonal anti-RTN3 (ThermoFisher Scientific, PA5–78316); mouse monoclonal anti-SERPINA1/AAT (Sigma-Aldrich, SAB4200198); rabbit polyclonal anti-Sec61A and Sec61B (Abcam, ab183046, ab15576); rabbit polyclonal anti-VCP (Huabio, ER30603); horseradish peroxidase (HRP)- conjugated goat anti-mouse IgG and anti-rabbit IgG (Jackson ImmunoResearch Laboratories, 115–035-003, 111–035-003).

### Cell lines.

Human embryonic kidney (HEK) 293 cell line transformed with SV40 large T antigen (HEK293T) and human cervical carcinoma cell line HeLa were purchased from American Type Culture Collection (ATCC, CRL-3216, CRM-CCL-2); human hepatoma cell line Huh7 was purchased from BioBW, China (BioBW, bio-73061); TZM-bI cells were from NIH HIV Reagent Program. All these cells were maintained in Dulbecco’s modi ed Eagle medium (DMEM; Thermo Fisher Scientific, 11965092) supplemented with 10% fetal bovine serum (FBS) and 1% penicillin-streptomycin (pen-strep; Thermo Fisher Scientific, 10378016) and cultivated at 37°C in the humidified atmosphere in a 5% CO_2_ incubator.

### CRISPR-Cas9 knockout.

DNA oligos encoding small guide RNAs (sgRNAs) targeting *MAN1B1*, *EDEM2*, *Membralin* (*TMEM259)*, *FAM134B*, *RTN3L*, *ATL3*, *SEC62*, *CCPG1, TEX264*, and *Sec61B* are shown in supplemental Table 1, which were cloned into the pSpCas9(BB)-2A-GFP (PX458) vector via BbsI digestion. HEK293T cells were transfected with these vectors, and after 48 h, single clones of GFP-positive cells were isolated by fluorescence-activated cell sorting. Knockout clones were identified by western blotting (WB) and further validated by DNA sequencing.

### siRNA knockdown.

*SEC61A* and *VCP* were knocked down by siRNAs using RNA oligo pairs 5’-CACUGAAAUGUCUACGUUUTT-3’/5’-AAACGUAGACAUUUCAGUGTT-3’, or 5’-GCAUGUGGGUGCUGACUUATT-3’/5’-UAAGUCAGCACCCACAUGCTT-3’. HEK293T cells were transfected with these siRNAs, and after 24 h, their expression was determined by WB.

### Plasmids.

The pcDNA3.1-hERManI-HA-FLAG, pCMV6-hEDEM1-Myc-FLAG, pcDNA3.1-hEDEM2-HA-FLAG, and pCMV6-hEDEM3-Myc-FLAG vectors and ERManI catalytically inactive mutant expression vector were reported previously ^6^. pcDNA3.1 vectors expressing ERManI/MAN1B1, EDEM1, EDEM2, and EDEM3 with a single C-terminal HA-tag were re-constructed in this study by homologous recombination after NheI/XhoI digestion. pNL-Luc-ΔEnv, pcDNA3.1-Flag-EBOV-GP, pcDNA3.1-FLAG-EBOV-GPΔMLD, pcDNA3.1-HiBiT-sGP, pcDNA3.1-HiBiT-ssGP, HIV-1 Env, and H5N1 HA expression vectors were reported previously ^8, 17^. pCAGGS-PDIA3-Myc, pCMV-CALR-Myc, pCMV6-CANX-Myc-FLAG, HSPA5/BiP promoter-luciferase reporter vector pLightSwitch-BiP, XBP1-activation reporter vector pXBP1u-FLuc, ATF4-expression reporter vector pATF4-UTR-Fluc, and ATF6-activation reporter vector p5xATF6-GL3 were reported previously ^8, 17^. pCMV-HA-Ub_WT_ and pCMV-HA-Ub_KO_ were reported previously ^37^. pSpCas9 (BB)-2A-GFP (PX458) was obtained from Feng Zhang through Addgene (48138). pCMV6-Membralin-Myc-Flag was ordered from Comatebio (CoME). Membralin deletion mutants (ΔTM1, ΔTM2, ΔTM3, ΔTM4, Δ4TM, 70–628, 1–322, 1–345, 1–466) were created by PCR and ASiSI/MluI digestion followed by homologous recombination. pCMV6-FAM134B-2-Myc-Flag was ordered from CoME. pCDNA3.1-FAM134B-Flag was ordered from GenScript. pCDNA3.1-NPC1–3xFlag were reported previously ^38^. pCDNA3.1-NHK was provided by Richard Sifers. pCMV6-SERPINA1-Myc-Flag, pCMV6-COL2A1-Myc-Flag, pCMV6-DYSF-Myc-Flag, pCMV6-CD3D-Myc-Flag, pCMV6-NCSTN-Myc-Flag, and pCMV6-VCP-Myc-FLAG were ordered from Origene (Cat# RC202082, RC218041, RC219485, RC210010, RC212646, RC211130). SERPINA1/AAT, NPC1, COL2A1, and DYSF mutants and pCMV6-VCP-His were created by PCR and ASiSI/MluI digestion followed by homologous recombination. All vectors constructed by us were confirmed by Sanger DNA sequencing. Detailed experimental procedures and primer sequences for the construction of these vectors are available upon request. Plasmids were prepared using Maxiprep kits (TIANGEN Biotech, DP117).

To express S proteins from SARS2, SARS1 and MERS, their cDNAs with a N-terminal FLAG tag were commercially synthesized and cloned into pCAGGS, resulting in pCAGGS-FLAG-SARS2-S, pCAGGGS-FLAG-SARS1-S, and pCAGGS-FLAG-MERS-S (Comate Bioscience, China). A SARS2-S and SARS1-S expression vector with a N-terminal Flag tag and C-terminal 19 amino acids deletion, pCAGGS-FLAG-SRAS2-S-D19 and pCAGGS-FLAG-SARS1-S-D19, were re-created by *EcoRI*/*XhoI* digestion. A SARS1-S expression vector with a C-terminal FLAG, pCAGGS-SARS1-S-FLAG, was re-created by *EcoRI*/*BspEI* digestion. These two SARS2-S and SARS1-S with this deletion were used to produce HIV-1 pseudovirions for infectivity assay. To express S protein-EGFP fusion proteins, these cDNAs were cloned into pEGFP-HA-N1 vector that express a HA-tag in front of EGFP by *XhoI*/*BspEI* digestion, resulting in pEGFP-SARS2-S-HA, pEGFP-SARS1-S-HA, pEFGP-MERS-S-HA, which express SARS2-S-HA-EGFP, SARS1-S-HA-EGFP, and MERS-S-HA-EGFP fusion proteins. To express MAN1B1-mCherry fusion, MAN1B1 was subcloned from pcDNA3.1-hMAN1B1-HA into pCAGGS-mCherry by *EcoRI* digestion. pEGFP-N1 vectors expressing CALR and TGLON2 were constructed by PCR and *XhoI*/*BspEI* digestion.

### Transfection.

HEK293T cells were cultured in 6-well plates and transfected with polyethyleneimine (PEI; Polysciences, 23966–2). HeLa cells were transfected using Lipofectamine 3000 according to the manufacturer’s protocol (Thermo Fisher Scientific, L3000015). The total indicated plasmids were diluted into 200μL serum-free Opti-MEM (Thermo Fisher Scientific, 31985062) and mixed with transfection reagents. After 20 min of incubation at room temperature, these transfection complexes were added directly into the supernatant of each well. Media were replaced after 6 h and cell lysate was collected at 48 h unless otherwise noted. Viruses released into the supernatants and proteins expressed in cells were analyzed (see below).

### Analysis of UPR.

HEK293T cells were cultured in 24-well plates and transfected with the indicated plasmid and reporter vectors as we reported previously ^6, 8^. HSPA5/BiP activation was measured by expressing BiP-RLuc reporter construct and measuring the intracellular Renilla luciferase activities. XBP1, ATF4, and ATF6 activation were measured by expressing XBP1-Fluc, pATF4-UTR-Fluc or p5×ATF6-GL3 reporter construct and measuring the intracellular Firefly luciferase activities. Luciferase activities were analyzed by Dual-Luciferase^®^ Reporter Assay System (Promega, E1910).

### Analysis of coronavirus spike protein-pseudotyped HIV-1 infectivity.

SARS2-S, SARS1-S, and MERS-S pseudotyped HIV-1 virions were produced from HEK293T WT and *MAN1B1*-KO cells as we reported previously ^38^. These cells were transfected with pNL-Luc-ΔEnv and a S protein expression vector in the presence or absence of a MAN1B1 expression. After 48 h, viruses were collected from the culture supernatants and clarified by low-speed centrifugation. After being normalized by p24^Gag^ ELISA ^39^, an equal amount of viruses were used to infect Huh7 cells. After 48 h of infection, cells were lysed, and viral infectivity was determined by measuring the intracellular luciferase activity using a firefly luciferase assay kit (US Everbright Inc, Cat# F6024).

### Western blotting (WB).

Transfected cells were lysed in RIPA buffer (25 mM Tris, pH7.4, 150 mM NaCl, 0.5% sodium deoxycholate, 0.1% SDS, 1%Nonidet P-40 [Sigma-Aldrich, R0278]) at 4°C. After centrifugation at 12,000 × g for 10 min at 4°C, cytosolic fractions were collected and boiled with SDS-polyacrylamide gel electrophoresis (SDS-PAGE) loading buffer (Solarbio Life Sciences, P1015). Proteins were separated by SDS-PAGE and transferred onto PVDF membranes. These membranes were blocked with 5% nonfat milk powder in TBST (Tris-buffered saline [20 mM Tris, pH 7.4,150 mM NaCl] containing 0.1% Tween 20; Solarbio Life Sciences, T8220) for 1 h at room temperature, and probed by primary antibodies followed by HRP-conjugated secondary antibodies. Chemiluminescence signals were then measured by incubating the membrane with SuperSignal substrate (Thermo Fisher Scientific, 34580).

### Immunoprecipitation.

After transfection of HEK293T cells cultured in a 6-cm dish, cells were lysed in 0.8 mL RIPA lysis buffer for 30 min on ice as we did previously ^40^. After the removal of nuclei via low-speed centrifugation and collecting 100 μL as input, the remaining 700 μL lysate was incubated with anti-FLAG M2 Magnetic beads (Sigma-Aldrich, M8823) and rotated at 4°C overnight. After being washed 3 times with 1 mL pre-cooled RIPA lysis buffer, proteins were removed from beads after boiling in 40 μL RIPA lysis buffer plus 15 μL sample loading buffer (5×) and analyzed by WB.

### In vivo polyubiquitination assay.

HEK293T cells were seeded in 6-cm dishes and transfected with vectors expressing SARS2-S and MAN1B1, or EBOV-GP and PDIA3, in the presence of ubiquitin expression vector as we did previously ^37^. After 48 h, cells in each dish were lysed in 600 μL RIPA buffer at 4°C for 30 min. After the removal of nuclei by low-speed centrifugation, 100 μL was collected as input, and the remaining 500 μL was incubated with anti-FLAG beads (Sigma-Aldrich, M8823) at 4°C overnight. Beads were washed three times with phosphate-buffered saline (PBS), then boiled in SDS-PAGE loading buffer and analyzed by WB.

### Confocal microscopy.

HeLa cells were seeded on a glass bottom cell culture dish (NEST Biotechnology, 801001) and transfected with various vectors using Lipofectamine 3000, as we did previously ^41^. After 30 h, cells were xed with 4% paraformaldehyde for 10 min, permeabilized with 0.1% Triton X-100 (Solarbio Life Sciences, T8200) for 10 min at room temperature, and then blocked with 5% bovine serum albumin (BSA; APPLYGEN, P1622) solution overnight at 4°C. Nuclei were stained with 4′,6-diamidino-2-phenylindole (DAPI) for 3–5 min and washed with PBS, and cells were observed and imaged under a confocal microscope (LSM880, Zeiss, White Plains, New York). Quantitative colocalization measurements were performed using ImageJ software. Pearson’s correlation coefficient (PCC) was calculated to describe the colocalization correlation of the intensity distributions between two channels.

### Live-cell imaging.

Approximately 1.0 ´ 10^5^ HEK293T cells were seeded on a glass bottom cell culture dish (NEST Biotechnology, 801001) and transfected with various vectors using PEI. After 14 h, Live-cell imaging was performed using LSM800 (Zeiss, White Plains, New York). During live-cell imaging, the dish was mounted in a chamber to maintain the incubation conditions at 37°C and 5% CO_2_.

### Statistical analysis.

All experiments were performed independently at least three times, with representative experiment being shown. GraphPad Prism (Graph Pad Software Inc., San Diego, CA, USA) was used for the data analysis. Data were presented as means ± standard error of measurements (SEMs) and represented by error bars. The significance of differences between samples was assessed by an unpaired two-tailed Student’s t test. A *p* value < 0.05 (*p* < 0.05) was statistically significant (*p < 0.05, **p < 0.01, ***p < 0.001), and *p* > 0.05 was not significant (ns).

## Supplementary Material

This is a list of supplementary files associated with this preprint. Click to download.

- SupplementalTableandFigures.docx
